# Recent advances in lignin valorization with bacterial cultures: microorganisms, metabolic pathways, and bio-products

**DOI:** 10.1186/s13068-019-1376-0

**Published:** 2019-02-15

**Authors:** Zhaoxian Xu, Peng Lei, Rui Zhai, Zhiqiang Wen, Mingjie Jin

**Affiliations:** 1 School of Environmental and Biological Engineering, Nanjing University of Science and Technology, Nanjing, 210094 China; 2 Nanjing Institute for Comprehensive Utilization of Wild Plants, Nanjing, 211111 China

**Keywords:** Biorefinery, Lignin valorization, Aromatic compounds, Metabolism of lignin-based aromatics, Biodegradation, Lignin-degrading bacteria, Lipid production, PHA production, *cis*, *cis*-muconic acid production

## Abstract

Lignin is the most abundant aromatic substrate on Earth and its valorization technologies are still under developed. Depolymerization and fragmentation are the predominant preparatory strategies for valorization of lignin to chemicals and fuels. However, due to the structural heterogeneity of lignin, depolymerization and fragmentation typically result in diverse product species, which require extensive separation and purification procedures to obtain target products. For lignin valorization, bacterial-based systems have attracted increasing attention because of their diverse metabolisms, which can be used to funnel multiple lignin-based compounds into specific target products. Here, recent advances in lignin valorization using bacteria are critically reviewed, including lignin-degrading bacteria that are able to degrade lignin and use lignin-associated aromatics, various associated metabolic pathways, and application of bacterial cultures for lignin valorization. This review will provide insight into the recent breakthroughs and future trends of lignin valorization based on bacterial systems.

## Background

**Fig. 1:**
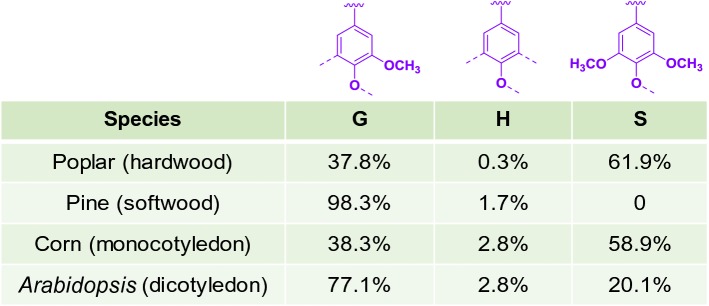
The basic structure and compositions of lignin units in different species. The lignin unit compositions mentioned here were quantified by 2D nuclear magnetic resonance technology [58]. H-lignin here may contain some residual 4-vinyl-phenol that comes from *p*-coumaric acid, which should not be included in “lignin”

Lignocellulose as the largest sustainable reservoir of organic material could be used to substitute for petroleum-based fuels and chemicals. Among the major components of lignocellulose, cellulose and hemicellulose have been converted to various chemicals and biofuels efficiently through biochemical route [1]. However, intense efforts are still needed to develop technologies to valorize lignin. Lignin, as a three-dimensional amorphous polymer, is composed of three different phenylpropane units: guaiacyl alcohol (G-type unit), *p*-coumaryl alcohol (H-type unit) and syringyl alcohol (S-type unit), which are linked mainly by aryl ether (*β*-O-4), phenylcoumaran (*β*-5), resinol (*β*–*β*), biphenyl ether (5-O-4), and dibenzodioxocin (5–5/*β*-O-4) [2, 3] (Fig. 1). Although lignin accounts for approximate 15%–40% of lignocellulose, it is the most underutilized fraction of lignocellulose [4, 5].

As mentioned by Nguyen et al. the second-generation biofuels began to be commercialized in 2015 and 67 facilities were operated throughout the world in 2017, with more than 1/3 of these operating at commercial scale [6]. In September 2017, fifteen ministries in China jointly issued an announcement to promote ethanol production for gasoline blending. The announcement stated that the large-scale production of cellulosic ethanol should be achieved in 2025. This suggests the continued growth in global cellulosic ethanol production. In cellulosic ethanol production process, hexose and pentose are fermented to ethanol, leaving most lignin in the solid residue. When 1.0 L cellulosic ethanol is produced, about 1.0 kg lignin will be generated as byproducts. Moreover, the global pulp/paper industry would also generate about 50 million tons of lignin annually. Although lignin is generated in large amount, valorization of lignin is still a great challenge due to its inherent structural heterogeneity [7, 8]. Currently, most lignin is mainly burned for energy supply or discarded into the environment. The life cycle assessment model indicated that valorizing partial lignin to target chemicals may be more environmentally beneficial than burning for energy supply only [9]. To make use of lignin more effectively, lignin valorization technologies are urgently desired.

With the aforementioned information, it is of great interest and challenge to realize the valorization of lignin. Although the structure of lignin is more complex compared with that of cellulose and hemicellulose, the high carbon/oxygen ratio and the abundant reserves of aromatic skeletal in lignin render it a promising feedstock for chemicals and fuels [10]. The predominant strategies for valorization of lignin to chemicals and fuels are depolymerization and fragmentation (ring scission), including reduction to modify lignin, supercritical fluids to modify lignin, ionic liquids to modify lignin, and fractionation by ultrafiltration and selective precipitation [10–13]. However, the structural heterogeneity of lignin generally results in diverse product species, which requires extensive separation and purification procedures [13–15].

Biological treatment is another choice for lignin valorization. In previous studies, the main biological treatment studies focused on wood-rotting basidiomycetes. Even though the investigation of lignin biodegradation by fungi has been carried out for decades and several progresses have been made, there are few commercial processes using fungi for lignin valorization.

Recently, bacterial systems have attracted an increasing attention on lignin valorization because of their inherent “biological funneling” processes, which can funnel multiple aromatic streams into a uniform compound [16]. Thus, the challenges associated with lignin heterogeneity can potentially be overcome. To summarize the current breakthroughs and discuss future trends of lignin valorization with bacterial systems, here the recent advances in lignin valorization with bacterial systems were reviewed and discussed, including the lignin-degrading bacteria and their screening methods, the lignin degradation pathways in bacteria, and the related bio-products produced from lignin components.

## The lignin-degrading bacteria and their screening methods

Discovery of bacteria with strong lignin degradation capability and characterization of related enzymes have significant benefits for lignin valorization. Sample source is a key factor for discovering lignin-degrading bacteria with excellent performance. The lignin-degrading bacteria are generally abundant in natural or manmade lignin-rich environments, such as leaf litter, sludge of pulp paper mill, compost soils, decomposing woods, and activated sludge. Some unusual samples also contain various lignin-degrading bacteria. For example, several lignin-degrading bacteria were separated from the steeping fluid of eroded bamboo slips, which were unearthed from the ancient tomb of more than 1700 years ago [17–19]. Wood-eating termites play an important role in natural carbon cycle and most lignocellulosic materials are digested in termite hindgut [20]. Thus, the termite gut is a rich source for the isolation of lignin-degrading bacteria and some bacteria responsible for lignin degradation were isolated successfully [21, 22]. In addition, some endophytes can decompose plant residues rapidly when the plants die due to its lignocellulose degradation ability, and endophytic bacteria were also isolated for lignin degradation [23].

In addition to sample sources, the screening method is another key factor for obtaining lignin-degrading bacteria with excellent performance. The commonly used method is enriching isolates by inoculating sample sources into mineral salts medium supplemented with lignin, synthetic lignin, or lignin-based aromatics as the sole carbon source [23–28]. In this case, the bacteria that cannot break down lignin and utilize lignin fragments for cell growth would be weeded out and lignin-degrading bacteria can be isolated. For instance, guaiacylglycerol-*β*-guaiacyl ether, a popular lignin model compound containing *β*-O-4 bonds, was applied to screen lignin degraders as *β*-O-4 linkages are the most abundant bonds in lignin [22, 29]. To inhibit the false positives caused by lignin degradation fungi, cycloheximide was commonly added into the selection medium [24, 30]. Even though the application of lignin and lignin-mimicked compounds can generally discover amounts of lignin-degrading bacteria, it cannot distinguish the lignin degradation abilities among the screened strains. Thus, it often entails a secondary screening. Due to the structural similarity between lignin fragments and some dyes, bacteria that can decolorize/degrade dyes generally have the capability to degrade lignin. Thus, the decoloration could serve as an index to determine the capabilities of lignin degradation. The commonly used dyes for lignin-degrading bacteria screening include Azure B, Toluidene Blue O, Methylene Blue, Malachite Green, Remazol Brilliant Blue R, indulin AT, etc. [21, 26, 28, 31–33].

For efficient screening of bacteria with exceptional lignin degradation capability, two high-throughput strategies were designed recently. In one case, target strains were screened based on their 2,2′-azino-bis(3-ethylbenzothiazoline-6-sulfonic acid) (ABTS) oxidizing activity. As well known, laccase belongs to ligninolytic enzyme system and most lignin-degrading bacteria can secrete laccase. ABTS can produce a green radical cation on oxidation by laccase, which has a strong absorption at 420 nm. Thus, when the culture broth of candidate bacteria was mixed with ABTS solution, the laccase-producing strains can be easily distinguished [29]. In another case, Chong and co-workers screened lignin degradation microbes based on the sensitive prussian blue spectrophotometric method [34]. When K_3_Fe(CN)_6_ and FeCl_3_ are mixed with phenolic hydroxyl groups, prussian blue is formed because of the redox reaction. By determining the absorbance of formed prussian blue at 710 nm, the lignin content in culture broth can be calculated [35, 36]. The culture broth with low lignin suggests the presence of bacteria with exceptional lignin degradation capability. The ABTS and prussian blue-based methods can be supervised with a spectrophotometric instrument; thus, it can be convenient to realize the high-throughput screening for lignin-degrading bacteria.

Based on the methods mentioned above, a considerable amount of lignin-degrading bacteria have been isolated. These bacteria mainly belong to phyla Proteobacteria, Actinobacteria, and Firmicutes. Recent study demonstrated that archaeal phylum *Bathyarchaeota* members also play an important role in lignin degradation [37]. The detailed information of the recently discovered lignin-degrading bacteria and typical lignin-degrading bacteria is shown in Table 1. More genotype and phenotype information associated with lignin-degrading bacteria can be found in the literature [38, 39]. With further researches, inherent enzymes and metabolic pathways involved in lignin degradation by these bacteria have been characterized, including enzymes/pathways catalyzing oxidative and hydroxylation reactions, depolymerizing phenolic and non-phenolic lignin polymers, demethylation reactions and opening the aromatic rings of lignin-based compounds [33, 40–45]. Now, some of these bacteria have been used in treatment of sewages from pulp and paper industry, degradation heterogeneous compounds, and pretreatment of lignocellulosic biomass [46–49]. In particular, among these isolates, some strains (e.g., *Pseudomonas putida* KT2440, *Sphingobium* sp. SYK-6, and *Rhodococcus opacus* PD630) have already been applied as typical cells for producing value-added products from lignin and mining new lignin-degrading enzymes. The detailed information will be elaborated in the following texts.

**Table 1:** The recently published lignin-degrading bacteria and their special characteristics

Taxonomic groups	Strains	Sample resources	Screen methods	Special characteristics	References
*α*-Proteobacteria	*Rhizobium* sp. YS-1r	Decaying biomass	Enrich bacteria in mineral salts medium supplemented with alkali lignin, and 0.02% cycloheximide	Possess nitrogen-fixing ability and aromatic hydrocarbons ability; possess a variety of lignin peroxidase and phenol oxidase	[24]
	*Novosphingobium* sp. B-7	Steeping fluid of eroded bamboo slips in ancient tomb	–	Can utilize kraft lignin as a sole carbon source; possess high manganese peroxidase activity and laccase activity	[19]
*β*-Proteobacteria	*Comamonas* sp. B-9	Steeping fluid of eroded bamboo slips in ancient tomb	–	Can reduce COD and color of lignin solutions; possess high manganese peroxidase activity and laccase activity	[17, 18]
	*Pandoraea norimbergensis* LD001 (DSM 24563)	Soil from beneath decomposing wood	Enrich bacteria in phosphate buffered mineral salts medium supplemented with kraft lignin	Can utilize at least seven lignin-based aromatic monomers; Can decolourize several lignin-mimicking dyes	[140]
*γ*-Proteobacteria	*P. deceptionensis* (DSM 105530)	Sediments from the Baltic Sea	Enrich bacteria in M9 medium supplemented with lignin-related compounds and cycloheximide	Can catabolize various aromatic compounds	[30]
	*Pseudomonas* sp. (DSM 104486)	Mature vegetable compost	Enrich bacteria in mineral salts medium with kraft pulping stream or technical kraft lignin as the sole carbon source	Can catabolize vanillin, vanillic acid, 4-hydroxybenzic acid, *p*-coumaric acid, benzoic acid, and ferulic acid	[141]
	*P. putida* NX-1	Leaf mold samples	Enrich bacteria in M9 medium supplemented with kraft lignin	Possess high lignin peroxidase activity, manganese peroxidase activity and laccase activity	[28]
	*Burkholderia* sp. strain CCA53	Soil samples from Higashi-Hiroshima	Enrich bacteria in mineral salts medium with alkali lignin as the sole carbon source	Can catabolize benzaldehyde, benzoic acid, catechol, 4-hydroxy benzaldehyde, 4-hydroxy benzoic acid, 4-hydroxybenzyl alcohol, syringol, and vanillin	[142]
	*Serratia* sp. JHT01 (DSM 29580), *Serratia liquefaciens* sp. PT01 (DSM 29581)	Forest surface soil	Primary screening with kraft lignin as the sole carbon source; secondary screening by determining the decolorization of Remazol Brilliant Blue R as indicator	Possess dye decolorization ability on Azure B, Methylene blue, Remazol Brilliant Blue R; Can catabolize syringaldehyde, syringic acid, vanillin, vanillic acid, vanillyl alcohol, guaiacol, veratyl alcohol, and biphenyl	[32]
	*Enterobacter* sp. PY12	Termites guts	Primary screening with lignin as the sole carbon source; secondary screening by determining the decolorization of Azure-B	Possess high lignin peroxides activity; possess dye decolorization ability on Congo red, Neutral red, Azure-B, Malachite green, and Methylene blue	[21]
	*Trabulsiella* sp. IIPTG13	Termite guts	Use guaiacylglycerol-*β*-guaiacyl ether as the sole carbon source	Can degrade lignin to organic acids and some low-molecular weight aromatics	[22]
	*Pantoea* sp. Sd-1	Rice seeds	Use lignin, rice straw powder, or lignin-related monomers as the sole carbon source	Can degrade both of cellulose, hemicellulose and lignin; can reduce color of lignin solutions	[23]
	*Enterobacter soli* sp. nov. LF7	Soil from the natural reserve	Enrich bacteria in mineral salts medium with alkali lignin as the sole carbon source	Possess high hydrogen producing ability; can produce the oxidizer of ABTS	[143]
	*Pseudomonas* sp. Q18	Rotten wood in forests	Enrich bacteria in mineral salts medium supplemented with lignin	Possess a novel bacterial DyP-type peroxidase;	[144]
Firmicutes	*Bacillus ligniniphilus* L1 (DSM 26145, JCM 18543)	Sediments from 3415 m depth of the South China Sea	Enrich bacteria in mineral salts medium with lignin as the sole carbon source	Can produce at least 15 kinds of aromatic compounds when using alkali lignin as the substrates	[40]
	*Acetoanaerobium* sp. WJDL-Y2	Anaerobic sludge from paper mill	Determine COD reduction of the broth containing lignin	It is an anaerobic bacterial strain; can reduce COD of lignin solutions	[145]
	*Aneurinibacillus aneurinilyticus*	Activated sludge from an effluent treatment plant of a pulp paper mill	Primary screening by determining color reduction of the broth containing lignin; secondary screening by using lignin-related low-molecular weight aromatics as substrates	Can reduce color and lignin content of lignin solutions; can degrade lignin to organic acids and some low-molecular weight aromatics	[27]
	*Paenibacillus glucanolyticus*	Black liquor	–	It is an anaerobic microorganism that can catabolize black liquor, cellulose, hemicellulose and lignin	[146]
	*Bacillus* sp. CS-1, *Bacillus* sp. CS-2	Forest soils of 0–15 cm depths	Determine the decolorization of Remazol Brilliant Blue R	Possess high laccase activity; can be used for biological pretreatment	[147]
	*Bacillus atrophaeus* LSSC3 and *Bacillus pumilus* strain CL29	Decayed plant material and soil in tropical rainforest	Determine isolates possessing laccase activity with ABTS as substrate	Can degrade guaiacylglycerol-*β*-guaiacyl ether	[29]
	*Bacillus* sp. SHC1	Soils of 0–10 cm depths from palm oil plantation	Primary screening by using kraft lignin as the sole carbon source; secondary screening by determining the decolorization of methylene blue	Possess high manganese peroxidase activity and lignin peroxidase activity	[33]
Actinobacteria	*Streptomyces* sp. Y-8	Soil samples	Enrich bacteria in mineral salts medium supplemented with lignin as the sole carbon source	Possess high laccase activity and lignin peroxidase activity	[148]
	*Rhodococcus opacus* PD630 (DSM 44193)	Soils from a gas-works plant	Enrich bacteria in mineral salts medium supplemented with phenyldecane as the sole carbon source	Can accumulate up to 87% (w/w) lipid in cell	[149]
	*Rhodococcus jostii* RHA1	Soils contaminated by *γ*-hexachlorocyclohexane	Enrich bacteria in mineral salts medium supplemented with biphenyl as the sole carbon source	Can break down some polychlorinated biphenyl congeners into smaller molecules	[150]
	*Rhodococcus pyridinivorans* CCZU-B16	Soil sample	Use a high-throughput screening strategy using Prussian blue spectrophotometric method	Can convert alkali lignin into microbial lipids	[34]

–, not mentioned in the paper

## Degradation pathways of lignin-based aromatics in bacteria

**Fig. 2:**
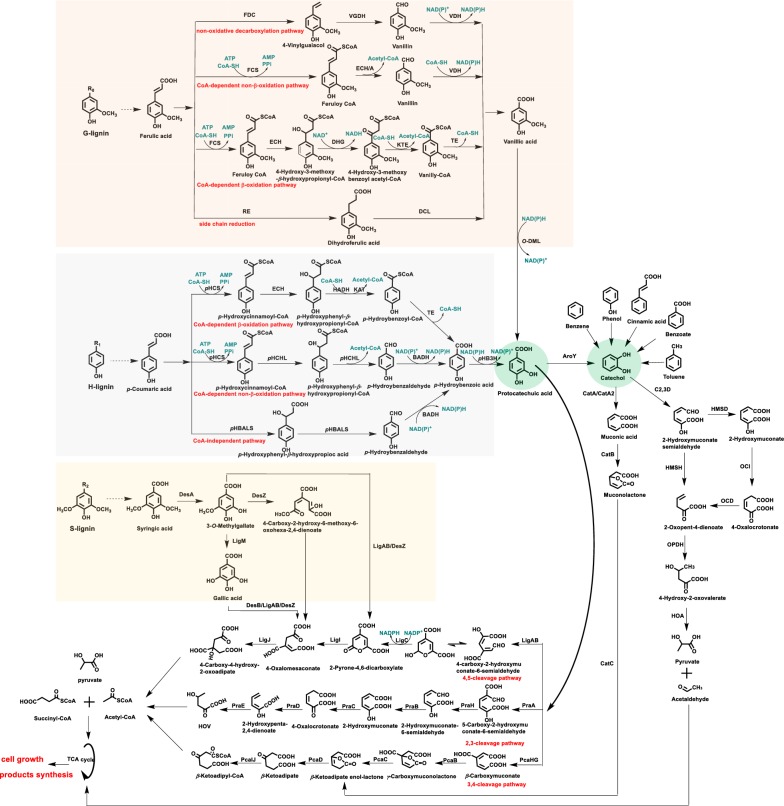
The scheme of degradation pathways for lignin-based aromatics (adapted from [4, 73, 95, 138, 139])

As mentioned above, bacteria have evolved multiple metabolic pathways to decompose lignin and assimilate its aromatic building blocks, and these sophisticated metabolic pathways are essential for lignin degradation. With the development of multi-omics technology, lignin-related metabolic pathways are becoming more accessible [40, 44, 45, 50]. Similar to fungi, some bacteria depolymerize lignin using multifarious enzymes, such as laccases, manganese peroxidases, dye-decolorizing peroxidases, cytochrome P450s, non-heme iron enzymes, dioxygenase, superoxide dismutases, and *β*-etherase enzymes. These lignin-degrading enzymes have been summarized in some excellent reviews [51–55]. Similarities and differences between bacteria and fungi in lignin depolymerization mechanism were described comprehensively in the above reviews. Even though the lignin depolymerization capability of bacteria is less than that of some famous lignin-degradation fungi [56], some of these bacteria can utilize the depolymerized lignin (lignin monomers, dimers and other low-molecular weight aromatic compounds) efficiently. It was hypothesized that lignin decomposition in nature is mainly initiated with fungi, which excrete powerful extracellular enzymes for lignin depolymerization. When lignin is depolymerized to monomers and/or low-molecular weight aromatics, bacteria assimilate them for carbon and energy through their well-adapted metabolic pathways [57]. This section focuses on the bacterial metabolic versatility for the assimilation of lignin-related low-molecular weight aromatics (Fig. 2).

### The degradation of G-lignin-based compounds (e.g., ferulic acid)

The G-lignin unit accounts for 37.8%, 98.3%, 38.3%, and 77.1% of the lignin in poplar (a typical hardwood) wood, pine (a typical softwood) wood, corn (a typical monocotyledon) stover, and *Arabidopsis* (a typical dicotyledon) inflorescence stem, respectively [58]. Ferulic acid is a standard model compound for G-lignin. Structurally, it is covalently linked to C-5 of the l-arabinofuranosyl residue which is attached to the xylan backbone and acts as an anchor of lignification in herbaceous biomass [59, 60]. As mentioned in “The lignin-degrading bacteria and their screening methods”, there are some bacteria that can degrade ferulic acid and utilize it as the sole carbon source for cell growth. The degradation pathway of ferulic acid can be divided into four categories: non-oxidative decarboxylation pathway [61, 62], coenzyme A (CoA)-dependent non-*β*-oxidation pathway [63–66], CoA-dependent *β*-oxidation pathway [66, 67], and side chain reduction pathway [68]. Even though involved with different intermediates and enzymes, these four pathways are all funneled into vanillic acid for further degradation. Moreover, some other lignin-based aromatics were also degraded through these pathways, such as vanillin, vanillic acid, and dihydroferulic acid (Fig. 2). In other words, these lignin intermediate products, if present in hydrolysates, can also be assimilated by these pathways.

### The degradation of *p*-coumaric acid

The H-lignin unit accounts for 0.3%, 1.7%, 2.8%, and 2.8% of the lignin in poplar wood, pine wood, corn stover, and *Arabidopsis* inflorescence stem, respectively [58]. It differs from G- and S-lignin units; there are no methoxy groups at the 3′ or 5′ positions of phenylpropane units. *p*-Courmaric acid and some other hydroxycinnamic acid have been commonly utilized to represent H-lignin units. Structurally, some *p*-coumaric acid is linked to the hemicellulose with ester linkages in herbaceous biomass, which can be released together with ferulic acid under alkaline hydrolysis conditions [59, 60, 69]. Generally, the degradation pathways of *p*-coumaric acid in bacteria can be divided into three categories: CoA-dependent *β*-oxidation pathway [70, 71], CoA-dependent non-*β*-oxidation pathway [64, 69, 72], and CoA-independent pathway [4, 73]. Ultimately, all these three pathways converge at the intermediate of *p*-hydroxybenzoic acid, which is then converted to protocatechuic acid by *p*-hydroxybenzoate-3-hydroxylase for further metabolism (Fig. 2).

### The degradation of S-lignin-based compounds (e.g., syringic acid)

The S-lignin unit accounts for 61.9%, 0, 58.9%, and 20.1% of the lignin in poplar wood, pine wood, corn stover, and *Arabidopsis* inflorescence stem, respectively [58]. There are two methoxy groups on the aromatic ring of S-lignin, in contrast to one and zero methoxy group on that of G-, and H-lignin, respectively, which make the degradation of S-lignin more difficult than that of G-, and H-lignin. Syringic acid is considered to be a model compound of S-lignin. Compared with ferulic acid and *p*-coumaric acid, there are fewer studies on microbes that can efficiently degrade syringic acid, which indicates the adverse effects of aromatic methoxy on S-lignin catabolism. The knowledge of bacterial S-lignin degradation pathway is mainly derived from *Sphingomonas* sp. SYK-6, which was isolated as a 5,5′-dehydrodivanillate degrader in 1987 [74]. Now, it is one of the most widely used bacteria for lignin degradation study.

In *Sphingomonas* sp. SYK-6 strain, syringic acid is first *O*-demethylated to 3-*O*-methylgallate (3MGA) by a tetrahydrofolate-dependent *O*-demethylase (DesA). Subsequently, there are three pathways for 3MGA degradation: gallic acid (GA) as an intermediate [75, 76], 4-carboxy-2-hydroxy-6-methoxy-6-oxohexa-2,4-dienoate as an intermediate [77, 78], 3MGA is directly integrated into protocatechuic acid 4,5-cleavage pathway [78, 79]. With the aforementioned three pathways, syringic acid is assimilated into the protocatechuic acid 4,5-cleavage pathway and ultimately integrated to the tricarboxylic acid (TCA) cycle (Fig. 2).

### The degradation of protocatechuic acid and catechol

Protocatechuic acid and catechol are widely distributed in various lignin hydrolysates and they are also two key branch points in bacterial lignin degradation pathways (Fig. 2). From the above discussion, both G- and H-lignin components are metabolized using protocatechuic acid as intermediate and S-lignin components are degraded through the protocatechuic acid 4,5-cleavage pathway. The degradation of protocatechuic acid in microbes has been classified into three categories: 3,4-cleavage pathway [80, 81], 4,5-cleavage pathway [82–84], and 2,3-cleavage pathway [85, 86]. Some other lignin-based aromatics (e.g., phenol, benzene, benzoate, toluene, naphthalene, etc.) could be funneled into catechol for further degradation. The degradation of catechol is mainly catalyzed by dioxygenases through *ortho*- or *meta*-cleavage pathway [87–92]. In nature, the *ortho*-cleavage pathway and *meta*-cleavage pathway of catechol are not exclusive to each other; they can co-exist in a bacterium. However, they may be induced differently by different carbon sources. For example, when grown on salicylic acid, only the catechol *ortho*-pathway is induced in *P. cepacia*. In contrast, when grown on benzoate, the *ortho*- and *meta*-pathways could be induced simultaneously for catechol degradation [93].

To summarize, bacteria have evolved a wide variety of distinct pathways to metabolize lignin-based compounds. The biochemical richness in bacteria provides great opportunities for pathway engineering for over-production of valuable intermediates. For example, the metabolisms of some G- and H-lignin compositions share protocatechuic acid as a common intermediate. Thus, these compounds can be funneled to various target products through protocatechuic acid.

## Application of multiple metabolic pathways for lignin valorization

Traditionally research on processing lignocellulose to biofuels and chemicals has focused on the carbohydrate fractions. However, as hexose and pentose fermentation technologies near maturation, it is becoming increasingly apparent that it is desirable to develop useful strategies to make use of considerable amount of residual lignin. However, the lignin components in fermentation streams are heterogeneous, containing organic acids, fermentation intermediates, and residual enzymes (e.g., cellulases) in addition to the targeted lignin-based compounds, which hinder its application in many fields (e.g., nanomaterials, fine chemicals). Biological conversion is a commendable supplement for the thermo-chemical lignin valorization route because some bacteria can funnel lignin fragments and other fermentation residuals into target chemicals [5, 11, 51, 94, 95]. Actually, several wild lignin-degrading bacteria possess lipid or polyhydroxyalkanoates (PHA) synthesis capability. Moreover, engineered bacteria with pyruvate, lactate, pyrogallol, and vanillin synthesis capability have also been constructed to utilize lignin and its fragments as substrates. All these examples demonstrate that it is technically feasible to convert lignin-enriched streams to value-added products with bacteria. In this section, recent advances in lignin conversion to bio-products are presented.

### Application of lignin degradation pathways for lipid production

Lipids are attractive feedstocks for production of biofuels. Generally, oleaginous organisms can accumulate > 20% of their dry cell weight (DCW) as lipids. In recent years, researchers found that some oleaginous microbes can synthesize lipid from lignin-based aromatics and *Rhodococcus* is such a promising species among these microbes, because of its robust growth, tolerance to various aromatics, broad substrate specificity, as well as robust lipid production capability [96, 97]. As mentioned in the “Degradation pathways of lignin-based aromatics in bacteria”, *Rhodococcus* bacteria can metabolize various lignin-based aromatics. These lignin-based aromatics undergo ring cleavage and are converted to acetyl-CoA, which is an essential precursor for lipid biosynthesis. For example, when *p*-hydroxybenzoic acid or vanillic acid was applied as the sole carbon source, both *R. opacus* PD630 and *R. opacus* DSM 1069 can survive well on these two lignin model monomers and accumulate approximately 20% of DCW as lipids under nitrogen limiting conditions [98]. Further studies demonstrate that blending lignin-based aromatics with glucose increased lipid contents considerably [99].

Compared with model aromatics, the actual residual lignin from biorefinery processes is of more interesting. Kosa and his co-workers validated the feasibility of lignin-to-lipid conversion with *R. opacus*, even though with limited lipid content and low lipid titer [100, 101] (Table 2). Monitoring lignin fractions during the fermentation process showed that the low-molecular weight lignin fractions were digested successfully, leaving the more recalcitrant lignin-based polymers undigested. It was suggested that these bacteria were not good at depolymerizing lignin, but can assimilate low-molecular weight lignin fractions efficiently. Thus, it was hypothesized that modifying lignin properties, such as decreasing the molecular weight and destructing obstinate chemical bonds, would be helpful for lipid accumulation with better efficiency. Thus, chemical lignin depolymerization methods and biological depolymerization methods were integrated with bacterial cultures for lipid production from lignin [35, 102]. In these ways, lignin was depolymerized first and then the depolymerized lignin can be utilized more efficiently by related bacteria. Moreover, bacteria co-culture systems were also applied for lipid production from lignin under the consideration that different bacteria can be complementary to each other on lignin depolymerization and assimilation [103].

**Table 2:** Lipid production from lignin degradation pathways by bacteria

Products	Substrates	Strains	Main Strategies	Titers	References
Lipid	4-Hydroxybenzoic acid, vanillic acid and glucose as the co-substrates	*Rhodococcus rhodochrous* ATCC 21198	Use lignin model monomer and glucose as co-substrates for lipid production	> 40% of DCW	[99]
	Ultrasonicated ethanol organosolv lignin	*R. opacus* DSM 1069	Integrate *R. opacus*-based assimilation process and ultrasonication-based lignin pretreatment process	4.08% of DCW	[101]
	O_2_ pretreated kraft lignin	*R. opacus* DSM 1069	Integrate *R. opacus*-based assimilation process and O_2_-based lignin pretreatment process	14.21% of DCW, 0.067 mg/mL	[102]
	Kraft lignin	*R. opacus* PD630	Integrate *R. opacus*-based assimilation process and laccase-based lignin depolymerization process	About 150 mg/L	[35]
	Alkali-extracted lignin	*R. opacus* PD630 and *R. jostii* RHA1 VanA^−^	Co-culture of *R. opacus* and *R. jostii* RHA1	0.39 g lipid/g DCW	[103]
	Algal hydrothermal liquefaction aqueous wastes	*R. opacus* PD630, *R. jostii* RHA1, and VanA^−^	Co-culture of *R. opacus* and *R. jostii* RHA1	0.46 g lipid/g DCW	[105]
	Biomass gasification wastewater	*R. opacus* DSM 43205	Supply wastewater with some mineral salt for better cell growth and lipid production	62.8% of DCW	[106]
	Effluent generated from a two-stage pretreatment of NaOH pre-extraction and alkaline H_2_O_2_ post-treatment.	*R. opacus* PD630	Integrate *R. opacus*-based assimilation process and alkali/alkali-peroxide-based pretreatment	1.3 g/L and 42.1% of DCW	[104]
	Ammonia fiber expansion corn stover lignin	*R. opacus* NRRL B-3311	Apply ammonia fiber expansion corn stover lignin without pretreatment for lipid production	32 mg/L	[151]
	Lignin from combinatorial pretreatment	*R. opacus* PD630	Consolidate combinatorial pretreatment, laccase addition and fed-batch fermentation processes	1.83 g/L	[152]

–, not mentioned in the paper

In addition to lignin residuals, aqueous wastes containing lignin-based aromatics can be also applied for lipid production with bacteria. A large amount of waste effluent could be generated during the pretreatment process of lignocellulose, which contains different fractions based on the pretreatment method. For example, alkali pretreatment processing would generate waste effluent that contains lignin, aromatic monomers, oligosaccharides, acetate, and other carbohydrates, which can be utilized by some lipid producing microorganisms [16, 103]. Thus, the co-production of bioethanol and lipid in an integrated biorefinery route was realized aimed at making use of all lignocellulose composition [104]. Thermo-chemical process is another choice for the conversion of lignocellulosic biomass to liquid fuels in addition to the biochemical process. In hydrothermal liquefaction processes, biomass is pyrolyzed to decompose the solid polymeric structure into a mixture of gas, biocrude, solid residue, and aqueous waste. Generally, this aqueous waste contains high concentrations of organic acids, alcohols, ketones, ammonia and lignin-based aromatics, which can be converted into lipids by some bacteria, along with the reduction of chemical oxygen demand (COD) [105]. Biomass gasification is another thermo-chemical process where biomass is converted to synthetic gas at a higher temperature. During the biomass gasification process, a large amount of wastewater is generated when the synthetic gas is scrubbed and it contains abundant lignin fractions. Using the biomass gasification wastewater with mineral salts as substrate, *R. opacus* DSM 43,205 can accumulate 62.8% g lipid/g DCW with a wastewater COD removal efficiency of 74% [106]. These two examples both indicated the prospect of bioconversion of underutilized aromatics in waste water from biorefinery process into useful products by selected bacteria.

### Application of lignin degradation pathways for PHA production

PHA is a group of biopolyesters synthesized as energy reserve inside cells and can be produced by variety of microbes under nutrient imbalance conditions. In the last three decades, PHA as biodegradable plastics has attracted wide attention not only because they have compatible material performance but also because they could be produced from renewable carbon sources, even from inferior biomass constituents [107]. As mentioned in “Application of lignin degradation pathways for lipid production”, lignin derivatives can be metabolized to acetyl-CoA, a precursor for lipid, as well as for PHA synthesis. PHA and lipid are all intracellular compounds, which can be separated from lignin streams just by centrifugation. Thus, it is a simpler process to convert lignin to PHA and lipid compared with pyrolyzing lignin to various chemicals which requires extensive separation and purification procedures.

There are various bacteria capable of producing PHA from lignin-based aromatics [16, 50, 108–111]. In addition to aromatic monomers, some actual lignin streams can also be utilized for PHA biosynthesis [112, 113] (Table 3). Just like lipid production from lignin-based streams, the PHA production from lignin streams by bacterial cultures is also generally concurrent with the reduction of the liquor color and COD [108, 114]. The PHA yield is relatively low when lignin residuals generated from traditional biorefinery process was used as substrates. As present in Table 3, only a milligram level of PHA was obtained when related bacteria were cultured in traditional lignin steams, such as kraft lignin and alkali-extracted lignin. Additional depolymerization steps can contribute to a better PHA production. Liu and co-workers applied lignin from an H_2_SO_4_ and NaOH combined pretreatment process as substrate for PHA production, and 1.0 g/L PHA was achieved. Further study indicated that this combined pretreatment process facilitated more lignin components accessible to PHA biosynthesis by increasing the contents of G- and H-lignin, reducing the *β*–*β* and *β*-O-4 bonds, and fractionating more aromatic monomers [115]. Besides integration of exogenous lignin depolymerization processes, enhancing the autologous lignin utilization capability of related bacteria is another choice for improving the lignin valorization efficiency. In this consideration, Lin and co-workers attempted to integrate three functional modules of lignin utilization in a wild *P. putida* strain, including the dye-decolorizing peroxidases-based lignin depolymerization system, the *β*-ketoadipate pathway-based aromatic compound catabolism system, and the PHA polymerase-based PHA synthesis system. As a result, this consolidated *P. putida* led to a sixfold increase of PHA titer [116]. To enable a broader slate of the produced PHA, the lignin valorization route was lengthened by consolidating the PHA production with a chemical catalysis. First, lignin stream or pretreated lignocellulosic liquor was converted to PHA by related microorganisms. Subsequently, the produced PHA was catalytically converted to alkenoic acids and hydrocarbons, which are precursors of diverse chemicals [16]. In this way, lignin can be transformed into biomaterials, chemical precursors and fuel-range hydrocarbons.

**Table 3:** PHA production from lignin degradation pathways by bacteria

Products	Substrates	Strains	Main strategies	Titers	Yields	References
PHA	Thermo-chemical wastewater streams	Engineered *P. putida* KT2440	Construct a strain with high tolerance to highly toxic substrates	–	–	[153]
	Lignin from a combined pretreatment strategy	Engineered *P. putida* KT2440	Perform a fed-batch fermentation and use lignin from a combined pretreatment strategy as substrate	1.0 g/L	17.6% mol/mol	[115]
	Kraft lignin	*Pandoraea* sp. ISTKB	Apply a nitrogen-limited culture condition	18 mg/L	–	[108]
	Kraft lignin	*Cupriavidus basilensis* B-8	Perform a fed-batch fermentation	319.4 mg/L	–	[114]
	Insoluble kraft lignin	Engineered *P. putida* A514	Strengthen three functional modules of lignin depolymerization system, aromatic compound catabolism system, and PHA synthesis system	75 mg/L		[116]
	Alkaline pretreated liquor	*P. putida* KT2440	Apply alkaline pretreated liquor directly without dilution	0.252 g/L	–	[16]
	Lignin	*Oceanimonas doudoroffii*	Perform a two-phase culture: the pre-culture with marine broth medium and the PHA production stage with mineral salt medium added lignin and lignin derivatives	0.2% of DCW	–	[154]
	benzoic acid	*P. putida* KT2440	Apply a nitrogen-limited culture condition	37.3% of DCW		[155]

–, not mentioned in the paper

### Application of lignin metabolic pathways for *cis, cis*-muconic acid production

The compound *cis, cis*-muconic acid (*cis, cis*-MA) attracts large amount of attention recently because it can be applied as an intermediate for adipic acid production, which is a bulk feedstock of fibers and plastics. There is a reported market greater than $22 billion for *cis, cis*-MA globally [117]. Currently, the industrial production of *cis, cis*-MA depends mainly on chemical synthesis using petroleum-based feedstocks. Compared with the widespread challenges of petrochemical processes, *cis, cis*-MA from renewable biomass provides a feasible alternative to alleviate the concerns of environmental issues and finite fossil resources. As presented in Fig. 2, *cis, cis*-MA is an intermediate of the *β*-ketoadipate pathway. Compared with lipid and PHA, *cis, cis*-MA is situated well before aromatics entering central carbon metabolism, which indicates that additional metabolic steps are not needed to divert aromatic carbons for cell growth and energy supply, and thus allows for a better atom efficiency.

As illustrated in Fig. 2, several aromatic-utilizing bacteria can employ catechol 1,2-dioxygenase to convert catechol to *cis, cis*-MA; however, *cis, cis*-MA is a metabolic intermediate and not accumulated by native strains. When *cis, cis*-MA degradation pathway was blocked, the recombinant strains can accumulate *cis, cis*-MA and secrete it into the culture broth. In native strains, phenol, benzene, benzoate, toluene, cinnamic acid and some other compounds, which wildly exist in lignin hydrolysate, can be funneled to *cis, cis*-MA through catechol. However, some other important components of lignin, such as *p*-coumaric acid, ferulic acid, and vanillin, were degraded using protocatechuic acid as a key intermediate, instead of catechol. Thus, these components of lignin cannot be converted to *cis, cis*-MA with the native metabolic pathways. To capture aromatics metabolized through protocatechuic acid degradation branch, Vardon and co-workers bridged the protocatechuic acid and catechol branches, along with blocking further metabolism of protocatechuic acid. Then, the modified strain was sequentially engineered to broaden its substrate spectrum (e.g., benzoate and phenol). Ultimately, the engineered strain could funnel multiple lignin-based aromatics and actual lignin steams to *cis, cis*-MA with a high efficiency [118] (Table 4). It is well known that almost all metabolic pathways are involved with multiple regulators as well as versatile key enzymes. The *cis, cis*-MA production pathway is no exception. The *cis, cis*-MA production capability was further improved by the co-expression of two genetically associated proteins of protocatechuic acid decarboxylase, and the deletion of the carbon catabolite repression control proteins [119, 120]. Recently, the titer, yield and productivity of MA from recombinant *P. putida* were constantly improved with a combination of gene overexpression, removal of global catabolic regulator, constant fed-batch and high-pH feeding strategy. As a result, as much as 50 g/L MA was produced from *p*-coumaric acid and 3.7 g/L *cis, cis*-MA was produced from base-catalyzed depolymerized lignin [121]. Although *cis, cis*-MA is secreted into the lignin streams during fermentation process compared with lipid and PHA, its structure of dicarboxylic acid is different to lignin-based aromatics. Thus, it can be also separated efficiently from lignin-based solutions. Based on the high *cis, cis*-MA titer, a separation and purification scheme consisting of protein removal process, active carbon cleanup process, crystallization process, and ethanol purification process was applied to achieve high purity *cis, cis*-MA (99.8%) [122]. The practical feasibility of the entire route from lignin to nylon-6,6 was demonstrated with the procedures of depolymerization of lignin, fed-batch fermentation process, recovery and purification of *cis, cis*-MA, catalytic hydrogenation, and polymerization of adipic acid to nylon 6,6 [123].

**Table 4:** *cis, cis*-MA production from lignin degradation pathways by bacteria

Products	Substrates	Strains	Main strategies	Titers	Yields	References
*cis, cis*-MA	Catechol	*C. glutamicum* MA-2	Delete *catB* gene; express *catA* gene; apply a fed-batch fermentation process	85 g/L	100% mol/mol	[125]
	*p*-Coumaric acid	*P. putida* KT2440-CJ103	Express *aroY* gene; delete *pcaHG* and *catB* genes; apply dissolved oxygen static fed-batch fermentation	13.5 g/L	–	[118]
	*p*-Coumaric acid	*P. putida* KT2440-CJ184	Co-express *ecdB* and *ecdD* with *aroY* gene	15.59 g/L	1.01 mol/mol	[120]
	*p*-Coumaric acid	*P. putida* KT2440-CJ238	Delete genes encoding carbon catabolite repression control protein	–	0.946 mol/mol	[119]
	*p*-Coumaric acid	*P. putida* KT2440-CJ242	Perform a fed-batch fermentation with by using high pH solution of *p*-coumaric acid as feeding solution	50 g/L	–	[121]
	Guaiacol	*Amycolatopsis* sp. ATCC 39116	Delete two putative *catB* genes; Perform a fed-batch fermentation	3.1 g/L	0.96 mol/mol	[124]
	Vanillin	Engineered *E. coli*	Co-express four genes of *vdh*, *desA*, *catA* and *aroY*	341 mg/L	0.69 g/g	[117]
	Sodium benzoate and glucose	*P. putida* KT2440-CJ102	Perform a dissolved oxygen-state fed-batch fermentation process	35.4 g/L	–	[122]
	Vanillin	Engineered *E. coli*	Co-express genes of *vdh*, *vanA*, *vanB*, *catA*, *aroY* and *kpdB*	–	–	[156]
	Lignin hydrolysate	*C. glutamicum* MA-2	Delete *catB* gene; express *catA* gene; apply a fed-batch fermentation process	1.8 g/L	–	[125]
	Alkaline pretreated liquor	*P. putida* KT2440-CJ103	Introduce *aroY* gene; delete *pcaHG* and *catB* genes	0.7 g/L	–	[118]
	Softwood lignin hydrolysate	*P. putida* IDPC/pTS110	Co-express *pcaHG* and *aroY* genes; apply a dissolved oxygen static batch fermentation process	–	0.3–0.331 mol/mol lignin-based aromatics	[126]
	Hardwood lignin hydrolysate	*Sphingobium* sp. SME257/pTS084	Use G-lignin components for *cis, cis*-MA production and S-lignin components for cell growth	26.8 mg/L	0.41 mol/mol birch lignin derivatives	[126]
	Softwood lignin hydrolysate	*P. putida* MA-9	Construct a strain with high tolerance to catechol; enhance catechol 1,2-dioxygenase expression levels; depolymerize softwood lignin in supercritical water	13 g/L	Nearly 100% from lignin-based aromatics	[123]
	Softwood lignin hydrolysate	*Amycolatopsis* sp. ATCC 39116	Delete two putative *catB* genes; pretreat lignin with low temperature hydrothermal conversion method	255.8 mg/L	0.72 mol/mol	[124]
	Alkaline pretreated lignin liquor	KT2440-CJ475	Perform a constant fed-batch fermentation process	0.65 g/L	> 100%^a^	[121]
	Base-catalyzed depolymerized lignin	KT2440-CJ242	Perform a constant fed-batch fermentation process	3.7 g/L	> 100%^a^	[121]

–, not mentioned in the paper

^a^The MA yield was calculated as MA mol/(*p*-coumaric acid + ferulic acid) mol

In addition to *P. putida*, other bacteria were also found to produce *cis, cis*-MA from lignin-based aromatics. *Amycolatopsis* sp. ATCC 39,116 with a deletion of two *cis*, *cis*-muconate cycloisomerases could accumulate 3.1 g/L *cis, cis*-MA from guaiacol with a yield of 96%, and 255.8 mg/L *cis, cis*-MA from softwood lignin hydrolysate [124]. In a very recently reported achievement, *Corynebacterium glutamicum* was also applied in *cis, cis*-MA production due to its robust tolerance to lignin-based aromatics. The recombinant *C. glutamicum* can produce 85 g/L and 1.8 g/L *cis, cis*-MA from catechol and lignin hydrolysate, respectively [125]. In the above studies, additional glucose or organic acid was needed for cell growth. Sonoki et al. constructed an engineered strain to utilize S-lignin derivatives for cell growth and G-lignin derivatives for *cis, cis*-MA production. This way, hardwood lignin which contained abundant G-lignin and S-lignin components could be utilized comprehensively for *cis, cis*-MA production without additional glucose [126]. As one of the most famous historic commercial microorganisms, *E. coli* was also applied for utilizing lignin-based aromatics because of its fast growth, unambiguous genetic background, and readily available genetic tools [127–129]. With different gene expression strategies, 100–314 mg/L *cis, cis*-MA were produced from vanillin [117].

### Application of lignin degradation pathways for aromatics

Since the European and US food legislations permitted the word “natural” to be used for products derived from biological sources, “natural” compounds have been considered superior and are more expensive than synthetic ones. Therefore, there is a growing interest in producing natural aromatics due to their wide applications. The unique aromatic structure and the reproducible characteristics of lignin make it an ideal feedstock for natural aromatics. However, vanillin, *p*-hydroxybenzoic acid, and pyrogallol are the only aromatics that have been produced from lignin through biological methods.

Vanillin is the main organoleptic ingredient of the vanilla pod and is widely applied by food, cosmetics, pharmaceutical, and other industries. Because of the limited supply and high price of natural vanillin, the current global demand for vanillin is mainly provided by chemical conversion of petrochemicals and thermo-chemical-mediated lignin degradation process [130]. Nowadays, consumers’ demands for natural products have motivated extensive research into biological methods for vanillin production from glucose, phenolic stilbenes, isoeugenol, eugenol, or ferulic acid through fungi, bacteria and plant cells [68]. There are a lot of microbes reported to have the capability of decomposing lignin to vanillin, including fungi and bacteria. However, vanillin was detected only in trace amounts in most cases. Recently, the vanillin metabolism in *R. jostii* RHA1 was blocked and the mutant strain was found to accumulate 96 mg/L vanillin when grown on 2.5% wheat straw lignocellulose [131] (Table 5). Just recently, the microbial fuel cell system has been also designed for lignin depolymerization to aromatics. In this case, lignin was dissolved in the aerobic cathode chamber and microbial electrochemical cells were cultivated in the anode chamber. When these two chambers were connected with a salt bridge and an external wire, electrons generated from microbial electrochemical cells will reduce oxygen molecules to produce H_2_O_2_ at the cathode. Then, lignin was depolymerized through H_2_O_2_-mediated oxidative reaction, with some vanillin produced [132]. It means that microbial fuel cell system may be a choice for the production of aromatics from lignin.

**Table 5:** Aromatic and other chemicals from lignin degradation pathways by bacteria

Products	Substrates	Strains	Main strategies	Titers	Yields	References
*p*-Hydroxybenzoic acid	*p*-Coumaric acid	Engineered *Burkholderia glumae* BGR1	Delete genes encoding *p*-hydroxybenzoate-3-hydroxylase, benzoyl-CoA ligase; overexpress *p*-hydroxcinnmaoyl-CoA synthetase II	2.73 g/L	99.0% mol/mol	[69]
Pyrogallol, GA	Syringic acid	Engineered *E. coli*	Co-express *desA*, *ligM* and a GA decarboxylase gene	7.3 mg/L pyrogallol, 18 mg/L GA	7.3 mg pyrogallol/g syringate, 18 mg GA/g syringate	[117]
Vanillin	2.5% Wheat straw lignocellulose	*R. jostii*	Delete *vdh* gene	96 mg/L	–	[131]
Vanillin	Lignin extracted from wheat straw	*Shewanella putrefaciens*	Construct a microbial fuel cell system for lignin depolymerization	275 mg/L	–	[132]
Pyruvate	*p*-Coumaric acid	*P. putida* KT2440-CJ122	Choose protocatechuic acid *meta*-cleavage pathway for *p*-coumaric acid conversation	–	0.414 g/g	[135]
Lactic acid	*p*-Coumaric acid	*P. putida* KT2440-CJ122	Choose protocatechuic acid *meta*-cleavage pathway for *p*-coumaric acid conversation	–	0.411 g/g	[135]
Pyruvate	*p*-Coumaric acid	*P. putida* KT2440-CJ124	Choose protocatechuic acid *ortho*-cleavage pathway for *p*-coumaric acid conversation	–	0.019 g/g	[135]
Lactic acid	*p*-Coumaric acid	*P. putida* KT2440-CJ124	Choose protocatechuic acid *ortho*-cleavage pathway for *p*-coumaric acid conversation	–	0.145 g/g	[135]
Methane	Hydrolysis lignin	Anaerobic microorganisms	Degrade lignin by anaerobic digestion in a nylon bag		125 mL methane/g volatile solid	[136]

–, not mentioned in the paper

*p*-Hydroxybenzoic acid is an important mono-hydroxybenzoic acid due to its excellent antimicrobial and antioxidant properties and low toxicity. Its esters are wildly used as preservatives in food, flavors, cosmetics and pharmaceutical products [133]. Although the synthetic technology for *p*-hydroxybenzoic acid has been developed for many years, there are still several limitations in the production of *p*-hydroxybenzoic acid by chemical synthesis, such as low regional selectivity, harsh conditions, and by-product formation. Jung and co-workers engineered a natural pathway to produce *p*-hydroxybenzoic acid by deleting genes involved in *p*-hydroxybenzoic acid degradation. With *p*-coumaric acid as substrate, the mutant strain accumulated 2.73 g/L *p*-hydroxybenzoic acid with a 99% conversion [69]. Pyrogallol is a common raw material used in chemical synthesis to produce bioactive molecules. Recent research work indicated that pyrogallol has benign anti-proliferative effects on some cancer cells [134]. Thus, there is a huge demand for pyrogallol, especially the biologically produced pyrogallol for medicine preparation. Wu and co-workers attempted to convert lignin-based aromatics into pyrogallol [117]. In their studies, two demethylase genes with a GA decarboxylase gene were co-expressed. The results showed that the recombinant strain yielded about 7.3 mg/L pyrogallol and 18 mg/L GA from syringic acid, which was obtained from hydrogen peroxide-catalyzed lignin.

In addition to the products mentioned above, lignin can also be converted to succinate, acetyl-CoA, pyruvate, and lactic acid through various metabolic pathways. Moreover, different aromatic metabolic pathways differ in intermediates, reducing equivalents, and carbon emitted, which will ultimately lead to different products, and/or different yields of the targeted product [135]. For example, the *ortho*-cleavage pathways of both protocatechuic acid and catechol yield one succinate and one acetyl-CoA; the *meta*-cleavage of catechol and the 2,3 *meta*-cleavage pathway of protocatechuic acid yield one pyruvate and one acetyl-CoA; the 4,5 *meta*-cleavage pathway of protocatechuic acid ultimately yields two pyruvates (Fig. 2).

The above chemicals are all produced by aerobic fermentation process. As mentioned in “The lignin-degrading bacteria and their screening methods”, some anaerobic bacteria can also metabolize lignin. These bacteria were also applied for lignin valorization. For example, hydrolysis lignin was converted into biogas by anaerobic digestion. In this way, the cellulose and hemicellulose parts of lignocellulose can be converted to ethanol and the lignin part can be converted to methane, which will improve the energy yield significantly [136]. With the aforementioned information, lignin represents a potential renewable feedstock for aromatics and other platform bio-products if suitable bio-catalysis routes are developed.

## Conclusion and perspectives

Even though various lignin-degrading bacteria were found widely and some lignin-based aromatics metabolic pathways have been elucidated and applied to produce bio-products. Based on the current knowledge, it has been suggested that the conversion of high-molecular weight lignin into bacteria available compounds (such as aromatic monomers or dimers) is the major bottleneck in the synthesis of bio-products from lignin. There is still a long way to go before lignin valorization at an industrial scale with bacteria can be realized technically and economically. The following perspectives should be considered for future studies:

1. Most lignin-degrading bacteria can only assimilate a fraction of lignin-based compounds. More efficient bacteria and metabolic pathways are in need for the comprehensive utilization of lignin or lignocellulosic biomass. As we further our understanding of lignin degradation process in bacteria, it is expected that pathway engineering can be applied in suitable bacterial hosts to assimilate more lignin components, as well as achieving high yields of the targeted products.
2. One drawback of lignin valorization by bacterial system is the low product titers. The product titers from lignin-based solutions are much lower than from glucose or other common substrates. Except the limited lignin utilization capability of target bacteria, the inhibition from lignin-based compounds is another key factor that causes the low microbial productivity. Fed-batch fermentation is a good solution for releasing the inhibition from high content lignin. Moreover, some microbes with high tolerance to lignin-based compounds can be obtained by screening, genetic engineering, or adapted evolution.
3. One major disadvantage of using microbes in lignin valorization processes is their low capability in utilizing water-insoluble and/or high-molecular weight lignin. Thus, appropriate depolymerization processes are required to disrupt lignin-enriched substrates into low-molecular weight and water-soluble species that can be assimilated by bacteria efficiently (Fig. 3). In particular, gasify the solid lignin and run a syngas bacterial culture may be a promising strategy for lignin valorization by bacteria cultures. In this case, the gasified components will be utilized more efficiently by related bacteria compared with the solid lignin or other lignin streams.

**Fig. 3:**
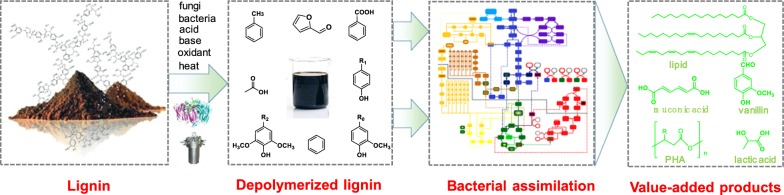
The scheme of hybrid lignin valorization route with depolymerization process and biochemical assimilation system
4. In previous lignocellulosic biorefinery designs, biomass pretreatment was generally designed for high content fermentable sugars. As different pretreatments can contribute to different lignin characteristics [137], lignin valorization is expected to be considered in addition to fermentable sugars when pretreatment and process are designed.
5. In addition to biological methods, other alternative methods have also applied wildly in lignin valorization, e.g., reduction, supercritical fluids, ionic liquids treatment, and fractionation by ultrafiltration and selective precipitation [11, 13]. These methods are not standalone and different alternatives pathways must be integrated for better lignin valorization. For example, the present results suggest that the productivities of lignin valorization by biological methods are relatively low and many bacteria can only utilize small lignin fragments. Thus, it is promising to integrate the chemical or physical lignin depolymerization processes and biological assimilation processes.

## Abbreviations

AroY: protocatechuic acid decarboxylase
CatA: catechol 1, 2-dioxygenase
CatB: *cis*, *cis*-muconate cycloisomerase
CatC: muconolactone isomerase
*cis, cis*-MA: *cis, cis*-muconic acid
CoA: coenzyme A
COD: chemical oxygen demand
DCW: dry cell weight
DesA: *O*-demethylase
GA: gallic acid
PcaHG: protocatechuic acid 3,4-dioxygenase
PHA: polyhydroxyalkanoates
PraA: protocatechuic acid 2,3-dioxygenase
TCA: tricarboxylic acid
VDH: vanillin dehydrogenase
3MGA: 3-*O*-methylgallate

## References

[1] Maity SK (2015). Opportunities, recent trends and challenges of integrated biorefinery: part I. Renew Sust Energ Rev..

[2] Vanholme R, Demedts B, Morreel K, Ralph J, Boerjan W (2010). Lignin biosynthesis and structure. Plant Physiol.

[3] Ralph J, Lundquist K, Brunow G, Lu F, Kim H, Schatz PF (2004). Lignins: natural polymers from oxidative coupling of 4-hydroxyphenyl-propanoids. Phytochem Rev.

[4] Chen Z, Wan C (2017). Biological valorization strategies for converting lignin into fuels and chemicals. Renew Sust Energy Rev..

[5] Ragauskas AJ, Beckham GT, Biddy MJ, Chandra R, Chen F, Davis MF (2014). Lignin valorization: improving lignin processing in the biorefinery. Science.

[6] Nguyen Q, Bowyer J, Howe J, Bratkovich S, Groot H, Pepke E, et al. Global production of second generation biofuels: trends and influences http://www.dovetailinc.org/report_pdfs/2017/dovetailbiofuels0117.pdf2017. Accessed 01 Jun 2017.

[7] Bruijnincx PCA, Rinaldi R, Weckhuysen BM (2015). Unlocking the potential of a sleeping giant: lignins as sustainable raw materials for renewable fuels, chemicals and materials. Green Chem.

[8] Wang H, Pu Y, Ragauskas A, Yang B (2019). From lignin to valuable products—strategies, challenges, and prospects. Bioresour Technol.

[9] Corona A, Biddy MJ, Vardon DR, Birkved M, Hauschild MZ, Beckham GT (2018). Life cycle assessment of adipic acid production from lignin. Green Chem.

[10] Rinaldi R, Jastrzebski R, Clough MT, Ralph J, Kennema M, Bruijnincx PC (2016). Paving the way for lignin valorisation: recent advances in bioengineering, biorefining and catalysis. Angew Chem Int Ed Engl.

[11] Schutyser W, Renders T, Van den Bosch S, Koelewijn SF, Beckham GT, Sels BF (2018). Chemicals from lignin: an interplay of lignocellulose fractionation, depolymerisation, and upgrading. Chem Soc Rev.

[12] Li C, Zhao X, Wang A, Huber GW, Zhang T (2015). Catalytic transformation of lignin for the production of chemicals and fuels. Chem Rev.

[13] Gillet S, Aguedo M, Petitjean L, Morais ARC, da Costa Lopes AM, Łukasik RM (2017). Lignin transformations for high value applications: towards targeted modifications using green chemistry. Green Chem.

[14] Lancefield CS, Ojo OS, Tran F, Westwood NJ (2015). Isolation of functionalized phenolic monomers through selective oxidation and C-O bond cleavage of the beta-O-4 linkages in lignin. Angew Chem Int Ed Engl.

[15] Toledano A, Serrano L, Balu AM, Luque R, Pineda A, Labidi J (2013). Fractionation of organosolv lignin from olive tree clippings and its valorization to simple phenolic compounds. Chemsuschem.

[16] Linger JG, Vardon DR, Guarnieri MT, Karp EM, Hunsinger GB, Franden MA (2014). Lignin valorization through integrated biological funneling and chemical catalysis. Proc Natl Acad Sci USA.

[17] Chai LY, Chen YH, Tang CJ, Yang ZH, Zheng Y, Shi Y (2014). Depolymerization and decolorization of kraft lignin by bacterium *Comamonas* sp. B-9. Appl Microbiol Biotechnol..

[18] Chen YH, Chai LY, Zhu YH, Yang ZH, Zheng Y, Zhang H (2012). Biodegradation of kraft lignin by a bacterial strain *Comamonas* sp. B-9 isolated from eroded bamboo slips. J Appl Microbiol..

[19] Chen Y, Chai L, Tang C, Yang Z, Zheng Y, Shi Y (2012). Kraft lignin biodegradation by *Novosphingobium* sp. B-7 and analysis of the degradation process. Bioresour Technol..

[20] Li H, Yelle DJ, Li C, Yang M, Ke J, Zhang R (2017). Lignocellulose pretreatment in a fungus-cultivating termite. Proc Natl Acad Sci USA..

[21] Zhou H, Guo W, Xu B, Teng Z, Tao D, Lou Y (2017). Screening and identification of lignin-degrading bacteria in termite gut and the construction of LiP-expressing recombinant Lactococcus lactis. Microb Pathog.

[22] Suman SK, Dhawaria M, Tripathi D, Raturi V, Adhikari DK, Kanaujia PK (2016). Investigation of lignin biodegradation by *Trabulsiella* sp. isolated from termite gut. Int Biodeterior Biodegrad..

[23] Xiong XQ, Liao HD, Ma JS, Liu XM, Zhang LY, Shi XW (2014). Isolation of a rice endophytic bacterium, *Pantoea* sp. Sd-1, with ligninolytic activity and characterization of its rice straw degradation ability. Lett Appl Microbiol..

[24] Jackson CA, Couger MB, Prabhakaran M, Ramachandriya KD, Canaan P, Fathepure BZ (2017). Isolation and characterization of *Rhizobium* sp. strain YS-1r that degrades lignin in plant biomass. J Appl Microbiol..

[25] Picart P, Wiermans L, Pérez-Sánchez M, Grande PM, Schallmey A, María P (2016). Assessing lignin types to screen novel biomass-degrading microbial strains: synthetic lignin as useful carbon source. ACS Sustain Chem Eng..

[26] Raj A, Reddy MM, Chandra R, Purohit HJ, Kapley A (2007). Biodegradation of kraft-lignin by *Bacillus* sp. isolated from sludge of pulp and paper mill. Biodegradation..

[27] Raj A, Chandra R, Reddy MMK, Purohit HJ, Kapley A (2007). Biodegradation of kraft lignin by a newly isolated bacterial strain, *Aneurinibacillus* *aneurinilyticus* from the sludge of a pulp paper mill. World J Microbiol Biotechnol.

[28] Xu Z, Qin L, Cai M, Hua W, Jin M (2018). Biodegradation of kraft lignin by newly isolated *Klebsiella pneumoniae*, *Pseudomonas putida*, and *Ochrobactrum tritici* strains. Environ Sci Pollut Res Int.

[29] Huang X-F, Santhanam N, Badri DV, Hunter WJ, Manter DK, Decker SR (2013). Isolation and characterization of lignin-degrading bacteria from rainforest soils. Biotechnol Bioeng.

[30] Ravi K, Garcia-Hidalgo J, Nobel M, Gorwa-Grauslund MF, Liden G (2018). Biological conversion of aromatic monolignol compounds by a *Pseudomonas* isolate from sediments of the Baltic Sea. AMB Express..

[31] Asina F, Brzonova I, Voeller K, Kozliak E, Kubatova A, Yao B (2016). Biodegradation of lignin by fungi, bacteria and laccases. Bioresour Technol.

[32] Tian JH, Pourcher AM, Peu P (2016). Isolation of bacterial strains able to metabolize lignin and lignin-related compounds. Lett Appl Microbiol.

[33] Rahman NHA, Rahman NAA, Aziz SA, Hassan MA (2013). Production of ligninolytic enzymes by newly isolated bacteria from palm oil plantation soils. BioResources.

[34] Chong GG, Huang XJ, Di JH, Xu DZ, He YC, Pei YN (2018). Biodegradation of alkali lignin by a newly isolated *Rhodococcus pyridinivorans* CCZU-B16. Bioprocess Biosyst Eng.

[35] Zhao C, Xie S, Pu Y, Zhang R, Huang F, Ragauskas AJ (2016). Synergistic enzymatic and microbial lignin conversion. Green Chem.

[36] Joshua CJ, Simmons BA, Singer SW (2016). Ferricyanide-based analysis of aqueous lignin suspension revealed sequestration of water-soluble lignin moieties. RSC Adv..

[37] Yu T, Wu W, Liang W, Lever MA, Hinrichs KU, Wang F (2018). Growth of sedimentary bathyarchaeota on lignin as an energy source. Proc Natl Acad Sci USA.

[38] Tian JH, Pourcher AM, Bouchez T, Gelhaye E, Peu P (2014). Occurrence of lignin degradation genotypes and phenotypes among prokaryotes. Appl Microbiol Biotechnol.

[39] Xu R, Zhang K, Liu P, Han H, Zhao S, Kakade A (2018). Lignin depolymerization and utilization by bacteria. Bioresour Technol.

[40] Zhu D, Zhang P, Xie C, Zhang W, Sun J, Qian WJ (2017). Biodegradation of alkaline lignin by *Bacillus ligniniphilus* L1. Biotechnol Biofuels.

[41] Sana B, Chia KH, Raghavan SS, Ramalingam B, Nagarajan N, Seayad J (2017). Development of a genetically programed vanillin-sensing bacterium for high-throughput screening of lignin-degrading enzyme libraries. Biotechnol Biofuels.

[42] Ma J, Zhang K, Liao H, Hector SB, Shi X, Li J (2016). Genomic and secretomic insight into lignocellulolytic system of an endophytic bacterium *Pantoea ananatis* Sd-1. Biotechnol Biofuels.

[43] Mallinson SJB, Machovina MM, Silveira RL, Garcia-Borras M, Gallup N, Johnson CW (2018). A promiscuous cytochrome P450 aromatic O-demethylase for lignin bioconversion. Nat Commun..

[44] Moraes EC, Alvarez TM, Persinoti GF, Tomazetto G, Brenelli LB, Paixao DAA (2018). Lignolytic-consortium omics analyses reveal novel genomes and pathways involved in lignin modification and valorization. Biotechnol Biofuels.

[45] Zhu D, Si H, Zhang P, Geng A, Zhang W, Yang B (2018). Genomics and biochemistry investigation on the metabolic pathway of milled wood and alkali lignin-derived aromatic metabolites of *Comamonas serinivorans* SP-35. Biotechnol Biofuels.

[46] Zhuo S, Yan X, Liu D, Si M, Zhang K, Liu M (2018). Use of bacteria for improving the lignocellulose biorefinery process: importance of pre-erosion. Biotechnol Biofuels.

[47] Hooda R, Bhardwaj NK, Singh P (2018). *Brevibacillus parabrevis* MTCC 12105: a potential bacterium for pulp and paper effluent degradation. World J Microbiol Biotechnol.

[48] Majumdar S, Priyadarshinee R, Kumar A, Mandal T, Dasgupta Mandal D (2019). Exploring *Planococcus* sp TRC1, a bacterial isolate, for carotenoid pigment production and detoxification of paper mill effluent in immobilized fluidized bed reactor. J Clean Prod..

[49] Bharagava RN, Mani S, Mulla SI, Saratale GD (2018). Degradation and decolourization potential of an ligninolytic enzyme producing *Aeromonas hydrophila* for crystal violet dye and its phytotoxicity evaluation. Ecotoxicol Environ Saf.

[50] Si M, Yan X, Liu M, Shi M, Wang Z, Wang S (2018). In situ lignin bioconversion promotes complete carbohydrate conversion of rice straw by *Cupriavidus basilensis* B-8. ACS Sustain Chem Eng..

[51] Bugg TD, Rahmanpour R (2015). Enzymatic conversion of lignin into renewable chemicals. Curr Opin Chem Biol.

[52] Brown ME, Chang MC (2014). Exploring bacterial lignin degradation. Curr Opin Chem Biol.

[53] Bugg TD, Ahmad M, Hardiman EM, Singh R (2011). The emerging role for bacteria in lignin degradation and bio-product formation. Curr Opin Biotechnol.

[54] Bugg TD, Ahmad M, Hardiman EM, Rahmanpour R (2011). Pathways for degradation of lignin in bacteria and fungi. Nat Prod Rep.

[55] de Gonzalo G, Colpa DI, Habib MH, Fraaije MW (2016). Bacterial enzymes involved in lignin degradation. J Biotechnol.

[56] Ahmad M, Taylor CR, Pink D, Burton K, Eastwood D, Bending GD (2010). Development of novel assays for lignin degradation: comparative analysis of bacterial and fungal lignin degraders. Mol BioSyst.

[57] Bugg TDH, Winfield CJ (1998). Enzymatic cleavage of aromatic rings: mechanistic aspects of the catechol dioxygenases and later enzymes of bacterial oxidative cleavage pathways. Nat Prod Rep.

[58] Mansfield SD, Kim H, Lu F, Ralph J (2012). Whole plant cell wall characterization using solution-state 2D NMR. Nat Protoc.

[59] Mussatto SI, Dragone G, Roberto IC (2007). Ferulic and p-coumaric acids extraction by alkaline hydrolysis of brewer’s spent grain. Ind Crops Prod.

[60] Jonsson LJ, Martin C (2016). Pretreatment of lignocellulose: formation of inhibitory by-products and strategies for minimizing their effects. Bioresour Technol.

[61] Mishra S, Sachan A, Vidyarthi AS, Sachan SG (2014). Transformation of ferulic acid to 4-vinyl guaiacol as a major metabolite: a microbial approach. Rev Environ Sci Bio..

[62] Kadakol JC, Kamanavalli CM (2010). Biodegradation *of eugenol by bacillus cere*us strain PN24. E J Chem.

[63] Yang W, Tang H, Ni J, Wu Q, Hua D, Tao F (2013). Characterization of two Streptomyces enzymes that convert ferulic acid to vanillin. PLoS ONE.

[64] Masai E, Harada K, Peng X, Kitayama H, Katayama Y, Fukuda M (2002). Cloning and Characterization of the Ferulic Acid Catabolic Genes of *Sphingomonas paucimobilis* SYK-6. Appl Environ Microbiol.

[65] Mitra A, Kitamura Y, Gasson MJ, Narbad A, Parr AJ, Payne J (1999). 4-hydroxycinnamoyl-CoA hydratase/lyase (HCHL)—an enzyme of phenylpropanoid chain cleavage from *Pseudomonas*. Arch Biochem Biophys.

[66] Plaggenborg R, Overhage J, Loos A, Archer JA, Lessard P, Sinskey AJ (2006). Potential of *Rhodococcus* strains for biotechnological vanillin production from ferulic acid and eugenol. Appl Microbiol Biotechnol.

[67] Gallage NJ, Moller BL (2015). Vanillin-bioconversion and bioengineering of the most popular plant flavor and its de novo biosynthesis in the vanilla orchid. Mol Plant..

[68] Priefert H, Rabenhorst J, Steinbüchel A (2001). Biotechnological production of vanillin. Appl Microbiol Biotechnol.

[69] Jung DH, Kim EJ, Jung E, Kazlauskas RJ, Choi KY, Kim BG (2016). Production of p-hydroxybenzoic acid from p-coumaric acid by *Burkholderia glumae* BGR1. Biotechnol Bioeng..

[70] Jung DH, Choi W, Choi KY, Jung E, Yun H, Kazlauskas RJ (2013). Bioconversion of p-coumaric acid to p-hydroxystyrene using phenolic acid decarboxylase from *B amyloliquefaciens* in biphasic reaction system. Appl Microbiol Biotechnol..

[71] Trautwein K, Wilkes H, Rabus R (2012). Proteogenomic evidence for beta-oxidation of plant-derived 3-phenylpropanoids in “*Aromatoleum aromaticum*” EbN1. Proteomics.

[72] Achterholt S, Priefert H, Steinbüchel A (2000). Identification of *Amycolatopsis* sp. strain HR167 genes, involved in the bioconversion of ferulic acid to vanillin. Appl Microbiol Biotechnol..

[73] Wang W, Zhang C, Sun X, Su S, Li Q, Linhardt RJ (2017). Efficient, environmentally-friendly and specific valorization of lignin: promising role of non-radical lignolytic enzymes. World J Microbiol Biotechnol.

[74] Katayama Y (1987). Cloning and expression of *Pseudomonas paucimobilis* SYK-6 genes involved in the degradation of vanillate and protocatechuate in *P. putida*. Mokuzai Gakkaishi..

[75] Kasai D, Masai E, Miyauchi K, Katayama Y, Fukuda M (2004). Characterization of the 3-O-methylgallate dioxygenase gene and evidence of multiple 3-O-methylgallate catabolic pathways in *Sphingomonas paucimobilis* SYK-6. J Bacteriol.

[76] Kasai D, Masai E, Miyauchi K, Katayama Y, Fukuda M (2005). Characterization of the gallate dioxygenase gene: three distinct ring cleavage dioxygenases are involved in syringate degradation by *Sphingomonas paucimobilis* SYK-6. J Bacteriol.

[77] Barry KP, Taylor EA (2013). Characterizing the promiscuity of LigAB, a lignin catabolite degrading extradiol dioxygenase from *Sphingomonas paucimobilis* SYK-6. Biochemistry.

[78] Kasai D, Masai E, Katayama Y, Fukuda M (2007). Degradation of 3-O-methylgallate in *Sphingomonas paucimobilis* SYK-6 by pathways involving protocatechuate 4,5-dioxygenase. FEMS Microbiol Lett.

[79] Masai E, Katayama Y, Fukuda M (2007). Genetic and biochemical investigations on bacterial catabolic pathways for lignin-derived aromatic compounds. Biosci Biotechnol Biochem.

[80] Yamanashi T, Kim SY, Hara H, Funa N (2015). In vitro reconstitution of the catabolic reactions catalyzed by PcaHG, PcaB, and PcaL: the protocatechuate branch of the beta-ketoadipate pathway in *Rhodococcus jostii* RHA1. Biosci Biotechnol Biochem.

[81] Harwood CS, Parales RE (1996). The β-ketoadipate pathway and the biology of self-identity. Annu Rev Microbiol.

[82] Ni B, Zhang Y, Chen DW, Wang BJ, Liu SJ (2013). Assimilation of aromatic compounds by *Comamonas testosteroni*: characterization and spreadability of protocatechuate 4,5-cleavage pathway in bacteria. Appl Microbiol Biotechnol.

[83] Kamimura N, Aoyama T, Yoshida R, Takahashi K, Kasai D, Abe T (2010). Characterization of the protocatechuate 4,5-cleavage pathway operon in *Comamonas* sp. strain E6 and discovery of a novel pathway gene. Appl Environ Microbiol..

[84] Kamimura N, Masai E. The protocatechuate 4,5-cleavage pathway: overview and new findings. Nojiri H, Tsuda M, Fukuda M, Kamagata Y, editors. Springer; 2014. p. 207–26.

[85] Kasai D, Fujinami T, Abe T, Mase K, Katayama Y, Fukuda M (2009). Uncovering the protocatechuate 2,3-cleavage pathway genes. J Bacteriol.

[86] Wolgel SA, Dege JE, Perkins-Olson PE, Jaurez-Garcia CH, Crawford RL, Münck E (1993). Purification and characterization of protocatechuate 2,3-dioxygenase from *Bacillus macerans*: a new extradiol catecholic dioxygenase. J Bacteriol.

[87] Vesely M, Knoppova M, Nesvera J, Patek M (2007). Analysis of catRABC operon for catechol degradation from phenol-degrading *Rhodococcus erythropolis*. Appl Microbiol Biotechnol.

[88] Ahmad SA, Shamaan NA, Syed MA, Khalid A, Ab Rahman NA, Khalil KA (2017). Meta-cleavage pathway of phenol degradation by *Acinetobacter* sp. strain AQ5NOL 1. Rendiconti Lincei..

[89] Mahiudddin M, Fakhruddin AN, Abdullah Al M (2012). Degradation of phenol via meta cleavage pathway by *Pseudomonas fluorescens* PU1. ISRN Microbiol..

[90] Kita A, Kita S-I, Fujisawa I, Inaka K, Ishida T, Horiike K (1999). An archetypical extradiol-cleaving catecholic dioxygenase: the crystal structure of catechol 2,3-dioxygenase (metapyrocatechase) from *Pseudomonas putida* mt. Structure..

[91] Kukor JJ, Olsen RH (1991). Genetic organization and regulation of a meta cleavage pathway for catechols produced from catabolism of toluene, benzene, phenol, and cresols by *Pseudomonas pickettii* PKO1. J Bacteriol..

[92] Hughes EJ, Bayly RC (1983). Control of catechol meta-cleavage pathway in *Alcaligenes eutrophus*. J Bacteriol.

[93] Hamzah RY, Al-Baharna BS (1994). Catechol ring-cleavage in *Pseudomonas cepacia*: the simultaneous induction of ortho and meta pathways. Appl Microbiol Biotechnol.

[94] Abdelaziz OY, Brink DP, Prothmann J, Ravi K, Sun M, Garcia-Hidalgo J (2016). Biological valorization of low molecular weight lignin. Biotechnol Adv.

[95] Seaton SC, Neidle EL. Lignin valorization: Royal Society of Chemistry; 2018.

[96] Shields-Menard SA, Amirsadeghi M, French WT, Boopathy R (2018). A review on microbial lipids as a potential biofuel. Bioresour Technol.

[97] Mahan KM, Le RK, Yuan J, Ragauskas AJ (2017). A review on the bioconversion of lignin to microbial lipid with oleaginous *Rhodococcus opacus*. J Biotechnol Biomater..

[98] Kosa M, Ragauskas AJ (2012). Bioconversion of lignin model compounds with oleaginous *Rhodococci*. Appl Microbiol Biotechnol.

[99] Shields-Menard SA, AmirSadeghi M, Green M, Womack E, Sparks DL, Blake J (2017). The effects of model aromatic lignin compounds on growth and lipid accumulation of *Rhodococcus rhodochrous*. Int Biodeterior Biodegrad..

[100] Kosa M. Direct and multistep conversion of lignin to biofuels: Georgia Institute of Technology; 2012.

[101] Kosa M, Ragauskas AJ (2013). Lignin to lipid bioconversion by oleaginous *Rhodococci*. Green Chem.

[102] Wei Z, Zeng G, Huang F, Kosa M, Huang D, Ragauskas AJ (2015). Bioconversion of oxygen-pretreated Kraft lignin to microbial lipid with oleaginous *Rhodococcus opacus* DSM 1069. Green Chem.

[103] He Y, Li X, Ben H, Xue X, Yang B (2017). lipid production from dilute alkali corn stover lignin by *Rhodococcus* strains. ACS Sustain Chem Eng..

[104] Le RK, Wells T, Das P, Meng X, Stoklosa RJ, Bhalla A (2017). Conversion of corn stover alkaline pre-treatment waste streams into biodiesel via *Rhodococci*. RSC Adv..

[105] He Y, Li X, Xue X, Swita MS, Schmidt AJ, Yang B (2017). Biological conversion of the aqueous wastes from hydrothermal liquefaction of algae and pine wood by *Rhodococci*. Bioresour Technol.

[106] Goswami L, Tejas Namboodiri MM, Vinoth Kumar R, Pakshirajan K, Pugazhenthi G (2017). Biodiesel production potential of oleaginous Rhodococcus opacus grown on biomass gasification wastewater. Renew Energy.

[107] Madkour MH, Heinrich D, Alghamdi MA, Shabbaj II, Steinbuchel A (2013). PHA recovery from biomass. Biomacromology.

[108] Kumar M, Singhal A, Verma PK, Thakur IS (2017). Production and Characterization of Polyhydroxyalkanoate from Lignin Derivatives by *Pandoraea* sp. ISTKB. ACS Omega..

[109] Tomizawa S, Chuah J-A, Matsumoto K, Doi Y, Numata K (2014). Understanding the limitations in the biosynthesis of polyhydroxyalkanoate (PHA) from lignin derivatives. ACS Sustain Chem Eng..

[110] Zhang Y, Wusiman A, Liu X, Wan C, Lee DJ, Tay J (2018). Polyhydroxyalkanoates (PHA) production from phenol in an acclimated consortium: batch study and impacts of operational conditions. J Biotechnol.

[111] Wang X, Lin L, Dong J, Ling J, Wang W, Wang H (2018). Simultaneous improvements of *Pseudomonas* cell growth and Polyhydroxyalkanoate production from a lignin derivative for lignin-consolidated bioprocessing. Appl Environ Microbiol..

[112] Salvachúa D, Karp EM, Nimlos CT, Vardon DR, Beckham GT (2015). Towards lignin consolidated bioprocessing: simultaneous lignin depolymerization and product generation by bacteria. Green Chem.

[113] Kumar P, Maharjan A, Jun HB, Kim BS (2018). Bioconversion of lignin and its derivatives into polyhydroxyalkanoates: challenges and opportunities. Biotechnol Appl Biochem..

[114] Shi Y, Yan X, Li Q, Wang X, Liu M, Xie S (2017). Directed bioconversion of Kraft lignin to polyhydroxyalkanoate by *Cupriavidus basilensis* B-8 without any pretreatment. Process Biochem..

[115] Liu Z-H, Olson ML, Shinde S, Wang X, Hao N, Yoo CG (2017). Synergistic maximization of the carbohydrate output and lignin processability by combinatorial pretreatment. Green Chem.

[116] Lin L, Cheng Y, Pu Y, Sun S, Li X, Jin M (2016). Systems biology-guided biodesign of consolidated lignin conversion. Green Chem.

[117] Wu W, Dutta T, Varman AM, Eudes A, Manalansan B, Loque D (2017). Lignin valorization: two hybrid biochemical routes for the conversion of polymeric lignin into value-added chemicals. Sci Rep..

[118] Vardon DR, Franden MA, Johnson CW, Karp EM, Guarnieri MT, Linger JG (2015). Adipic acid production from lignin. Energy Environ Sci.

[119] Johnson CW, Abraham PE, Linger JG, Khanna P, Hettich RL, Beckham GT (2017). Eliminating a global regulator of carbon catabolite repression enhances the conversion of aromatic lignin monomers to muconate in Pseudomonas putida KT2440. Metab Eng Commun.

[120] Johnson CW, Salvachúa D, Khanna P, Smith H, Peterson DJ, Beckham GT (2016). Enhancing muconic acid production from glucose and lignin-derived aromatic compounds via increased protocatechuate decarboxylase activity. Metab Eng Commun.

[121] Salvachúa D, Johnson CW, Singer CA, Rohrer H, Peterson DJ, Black BA (2018). Bioprocess development for muconic acid production from aromatic compounds and lignin. Green Chem.

[122] Vardon DR, Rorrer NA, Salvachúa D, Settle AE, Johnson CW, Menart MJ (2016). cis, cis-muconic acid: separation and catalysis to bio-adipic acid for nylon-6,6 polymerization. Green Chem.

[123] Kohlstedt M, Starck S, Barton N, Stolzenberger J, Selzer M, Mehlmann K (2018). From lignin to nylon: cascaded chemical and biochemical conversion using metabolically engineered *Pseudomonas putida*. Metab Eng..

[124] Barton N, Horbal L, Starck S, Kohlstedt M, Luzhetskyy A, Wittmann C (2018). Enabling the valorization of guaiacol-based lignin: integrated chemical and biochemical production of cis, cis-muconic acid using metabolically engineered *Amycolatopsis* sp. ATCC 39116. Metab Eng.

[125] Becker J, Kuhl M, Kohlstedt M, Starck S, Wittmann C (2018). Metabolic engineering of *Corynebacterium glutamicum* for the production of cis, cis-muconic acid from lignin. Microb Cell Fact.

[126] Sonoki T, Takahashi K, Sugita H, Hatamura M, Azuma Y, Sato T (2017). Glucose-free cis, cis-muconic acid production via new metabolic designs corresponding to the heterogeneity of lignin. ACS Sustain Chem Eng..

[127] Clarkson SM, Giannone RJ, Kridelbaugh DM, Elkins JG, Guss AM, Michener JK (2017). Construction and optimization of a heterologous pathway for protocatechuate catabolism in *Escherichia coli* enables bioconversion of model aromatic compounds. Appl Environ Microbiol..

[128] Wu W, Liu F, Singh S (2018). Toward engineering *E. coli* with an autoregulatory system for lignin valorization. Proc Natl Acad Sci USA.

[129] Varman AM, Follenfant R, Liu F, Davis RW, Lin YK, Singh S (2018). Hybrid phenolic-inducible promoters towards construction of self-inducible systems for microbial lignin valorization. Biotechnol Biofuels..

[130] Fache M, Boutevin B, Caillol S (2015). Vanillin production from lignin and its use as a renewable chemical. ACS Sustain Chem Eng..

[131] Sainsbury PD, Hardiman EM, Ahmad M, Otani H, Seghezzi N, Eltis LD (2013). Breaking down lignin to high-value chemicals: the conversion of lignocellulose to vanillin in a gene deletion mutant of *Rhodococcus jostii* RHA1. ACS Chem Biol.

[132] Sharma RK, Mukhopadhyay D, Gupta P (2018). Microbial Fuel cell mediated lignin depolymerization: a sustainable approach. J Chem Technol Biotechnol..

[133] Soni MG, Carabin IG, Burdock GA (2005). Safety assessment of esters of p-hydroxybenzoic acid (parabens). Food Chem Toxicol.

[134] Nemec MJ, Kim H, Marciante AB, Barnes RC, Talcott ST, Mertens-Talcott SU (2016). Pyrogallol, an absorbable microbial gallotannins-metabolite and mango polyphenols (*Mangifera Indica* L.) suppress breast cancer ductal carcinoma in situ proliferation in vitro. Food Funct..

[135] Johnson CW, Beckham GT (2015). Aromatic catabolic pathway selection for optimal production of pyruvate and lactate from lignin. Metab Eng.

[136] Mulat DG, Dibdiakova J, Horn SJ (2018). Microbial biogas production from hydrolysis lignin: insight into lignin structural changes. Biotechnol Biofuels.

[137] Narron RH, Kim H, Chang HM, Jameel H, Park S (2016). Biomass pretreatments capable of enabling lignin valorization in a biorefinery process. Curr Opin Biotechnol.

[138] Kamimura N, Takahashi K, Mori K, Araki T, Fujita M, Higuchi Y (2017). Bacterial catabolism of lignin-derived aromatics: new findings in a recent decade: Update on bacterial lignin catabolism. Environ Microbiol Rep..

[139] Nesvera J, Rucka L, Patek M. Adv Appl Microbiol. Academic Press; 2015. p. 107–60. 10.1016/bs.aambs.2015.06.002 26505690

[140] Bandounas L, Wierckx NJ, Winde JH, Ruijssenaars HJ (2011). Isolation and characterization of novel bacterial strains exhibiting ligninolytic potential. BMC Biotechnol..

[141] Ravi K, Garcia-Hidalgo J, Gorwa-Grauslund MF, Liden G (2017). Conversion of lignin model compounds by *Pseudomonas putida* KT2440 and isolates from compost. Appl Microbiol Biotechnol.

[142] Akita H, Kimura Z, Mohd Yusoff MZ, Nakashima N, Hoshino T (2016). Isolation and characterization of *Burkholderia* sp. strain CCA53 exhibiting ligninolytic potential. Springerplus..

[143] Manter DK, Hunter WJ, Vivanco JM (2011). *Enterobacter soli* sp. nov.: a lignin-degrading gamma-proteobacteria isolated from soil. Curr Microbiol..

[144] Yang C, Yue F, Cui Y, Xu Y, Shan Y, Liu B (2018). Biodegradation of lignin by *Pseudomonas* sp Q18 and the characterization of a novel bacterial DyP-type peroxidase. J Ind Microbiol Biotechnol..

[145] Duan J, Huo X, Du WJ, Liang JD, Wang DQ, Yang SC (2016). Biodegradation of kraft lignin by a newly isolated anaerobic bacterial strain, *Acetoanaerobium* sp. WJDL-Y2. Lett Appl Microbiol..

[146] Mathews SL, Pawlak JJ, Grunden AM (2014). Isolation of *Paenibacillus glucanolyticus* from pulp mill sources with potential to deconstruct pulping waste. Bioresour Technol.

[147] Chang YC, Choi D, Takamizawa K, Kikuchi S (2014). Isolation of *Bacillus* sp. strains capable of decomposing alkali lignin and their application in combination with lactic acid bacteria for enhancing cellulase performance. Bioresour Technol..

[148] Zhou G, Zhuang X, Yuan Z, Tan X, Qi W, Yu Q (2016). Isolation of *Streptomyces* sp. strains capable of degrading lignin under alkaline conditions and its degradation properties. J Biobased Mater Bio..

[149] Alvarez HM, Mayer F, Fabritius D, Steinbüchel A (1996). Formation of intracytoplasmic lipid inclusions by *Rhodococcus opacus* strain PD630. Arch Microbiol.

[150] Seto M, Kimbara K, Shimura M, Hatta T, Fukuda M, Yano K (1995). A novel transformation of polychlorinated biphenyls by *Rhodococcus* sp. strain RHA1. Appl Environ Microbiol..

[151] Wang Z, Li N, Pan X (2019). Transformation of ammonia fiber expansion (AFEX) corn stover lignin into microbial lipids by *Rhodococcus opacus*. Fuel.

[152] Liu ZH, Xie S, Lin F, Jin M, Yuan JS (2018). Combinatorial pretreatment and fermentation optimization enabled a record yield on lignin bioconversion. Biotechnol Biofuels.

[153] Jayakody LN, Johnson CW, Whitham JM, Giannone RJ, Black BA, Cleveland NS (2018). Thermochemical wastewater valorization via enhanced microbial toxicity tolerance. Energy Environ Sci.

[154] Numata K, Morisaki K (2015). Screening of marine bacteria to synthesize polyhydroxyalkanoate from lignin: contribution of lignin derivatives to biosynthesis by *Oceanimonas doudoroffii*. ACS Sustain Chem Eng..

[155] Xu Z, Li X, Hao N, Pan C, Torre L, Ahamed A (2018). Kinetic understanding of nitrogen supply condition on biosynthesis of polyhydroxyalkanoate from benzoate by *Pseudomonas putida* KT2440. Bioresour Technol..

[156] Sonoki T, Morooka M, Sakamoto K, Otsuka Y, Nakamura M, Jellison J (2014). Enhancement of protocatechuate decarboxylase activity for the effective production of muconate from lignin-related aromatic compounds. J Biotechnol..

